# Dominant Negative Effect of Mutated Thyroid Stimulating Hormone Receptor (P556L) Causes Hypothyroidism in C.RF-Tshr^hyt/wild^ Mice

**DOI:** 10.1371/journal.pone.0042358

**Published:** 2012-08-16

**Authors:** Toyoshi Endo, Tetsuro Kobayashi

**Affiliations:** The Third Department of Internal Medicine, Interdisciplinary Graduate School of Medicine and Engineering, University of Yamanashi, Chuo City, Yamanashi, Japan; Cardiff University, United Kingdom

## Abstract

C.RF-Tshr^hyt/hyt^ mice have a mutated thyroid stimulating hormone receptor (P556L-TSHR) and these mice develop severe hypothyroidism. We found that C.RF-Tshr^hyt/wild^ heterozygous mice are also in a hypothyroid state. Thyroid glands from C.RF-Tshr^hyt/wild^ mice are smaller than those from wild-type mice, and ^125^I uptake activities of the former are significantly lower than those in the latter. When TSHR (TSHR(W)) and P556L-TSHR (TSHR(M)) cDNAs were cloned and co-transfected into HEK 293 cells, the cells retained ^125^I-TSH binding activity, but cAMP response to TSH was decreased to about 20% of HEK 293 cells transfected with TSHR(W) cDNA. When TSHR(W) and TSHR(M) were tagged with eCFP or eYFP, we observed fluorescence resonance energy transfer (FRET) in HEK 293 cells expressing TSHR(W)-eCFP and TSHR(W)-eYFP in the absence of TSH, but not in the presence of TSH. In contrast, we obtained FRET in HEK 293 cells expressing TSHR(W)-eCFP and TSHR (M)-eYFP, regardless of the presence or absence of TSH. These results suggest that P556L TSHR has a dominant negative effect on TSHR(W) by impairing polymer to monomer dissociation, which decreases TSH responsiveness and induces hypothyroidism in C.RF-Tshr^hyt/wild^ mice.

## Introduction

The thyroid stimulating hormone receptor (TSHR) is a member of the G protein-coupled family of receptors, whose main function is to regulate hormone synthesis, secretion and cell growth in thyroid glands [1].

TSHR knockout (TSHR-KO) mice have small thyroid glands, and show severe hypothyroidism with no detectable thyroid hormone and elevated TSH [2]. KO mice die within 1 week of weaning, unless fed a diet supplemented with thyroid hormone. In contrast, heterozygotes of TSHR-KO mice have normal circulating thyroid hormone and TSH levels, and are apparently unaffected.

C.RF-Tshr^hyt/hyt^ mice (Tshr^hyt/hyt^ mice) represent another model of hypothyroidism resulting from a TSHR mutation in the fourth transmembrane domain. A proline to leucine mutation at codon 556 results in plasma membrane targeting, but defective TSH binding and receptor function [3], [4]. Furthermore, it has been reported that the mutation did not alter TSHR expression levels [3].

At present, TSHR is detectable in a variety of cell types, including the adipose tissues [5], bone [6], [7], thymus heart, lymphocytes and retro-orbital fibroblasts [1]. Using Tshr^hyt/hyt^ mice, we previously reported that TSHR in brown adipose tissue is involved in the regulation of thermogenesis [8]. In the course of these studies, we found that Tshr^hyt/wild^ mice were also in a hypothyroid state. As heterozygotic TSHR KO mice are unaffected and maintain a euthyroid state, the evidence prompted us to study the reasons why Tshr^hyt/wild^ mice exhibit hypothyroidism.

## Materials and Methods

### Animals and Cells

All studies were approved by the Animal Research Committee of the University of Yamanashi. C.RF-Tshr^hty/wild^ mice were obtained from The Jackson Laboratory (Bar Harbor, ME), and were bred to generate experimental animals. Mice were kept in an SPF mouse room void of thyroid hormone supplementation. All mice were aged 70–84 days at the start of the experiments. Determination of TSHR genotype was carried out as described previously [8]. Rectal temperature was measured using a digital thermometer (TD-300; Shibaura Electronics Co., Ltd., Tokyo, Japan).

Human embryonic kidney cells 293 (HEK 293) (ECACC No. 85120602) were obtained from DS Pharma Biomedical Co. Ltd., Osaka, Japan), and were cultured in minimum essential medium (MEM) containing 10% fetal calf serum. Mammalian expression plasmids used were transfected into cells with a Gene Pulser (Xcell; BioRad Japan, Tokyo, Japan).

### Plasmid construction

In order to obtain wild-type and mutated mouse TSHR, we prepared mRNAs from thyroid glands of Tshr^hyt/wild^ mice, and reverse transcribed these mRNAs into cDNAs. Using the cDNAs as a template, PCR was carried out with the sense primer, 5′-ATCGCGGATCCGAAGTAGCC CAGAGGGTCC-3′, and the antisense primer, 5′-TCCGGAATTCTTACAAGGCTGTTTGCT TATACTC-3′. PCR products were digested with EcoRI and BamH1, and then ligated into the cloning site of pBluescript SK (Stratagene, LaJolla CA). Sequencing of codon 556 was carried out with the antisense primer, 5′-GTAGATCTTCACATAGCAGG-3′, and the receptor cDNAs were cloned into pcDNA3.1 (Invitrogen Co., San Diego, CA) to construct pcDNA-TSHR(wild, W) and pcDNA-TSHR(mutant, M). To prepare pcDNA-TSHR-eCFP or eYFP, we initially amplified cDNAs of these living color proteins having an EcoRI site at the 5′-end and a Not I site at the 3′-end by PCR using the sense primer, 5′-GTCAGAATTCATGGTGAGCAAGGGC GAGGAGCTG-3′, and the antisense primer, 5′-AGAGTCGCGGCCGCTTTACTTGTACAGC TCGTCCATGCC -3′, with pECFP or pEYFP (BD Clonthch Labo. Inc., Palo Alto, CA) as templates. After digesting PCR products with EcoR I and Not I, cDNAs were ligated into the cloning sites of pcDNA3.1 (pcDNA-eCFP and pcDNA-eYFP). TSHR(W) and TSHR(M) cDNAs lacking stop codons were amplified by PCR using the sense primer, 5′-ATCGCGGATC CGAAGTAGCCCAGAGGGTCC-3′, and the antisense primer, 5′-TCCGGAATTCCAAGGCT GTTTGCTTATACTCTTC-3′. TSHR(W) and TSHR(M) were ligated into pcDNA-eCFP and pcDNA-eYFP, respectively.

### Fluorescence resonance energy transfer (FRET)

HEK 293 cells (10^6^ cells) were transfected with 0.5 µg of pcDNA-TSHR(W or M)-eCFP and the same amount of pcDNA-TSHR(W or M)-eYFP by electroporation. After 72 h of culture, using eCFP as a donor (D) and eYFP as an acceptor (A), emission FRET studies in regions of interests (RoI) in single living cell were carried out with or without 1 mU/ml TSH using an Olympus IX80 microscope equipped with emission and excitation FRET filter wheels controlled by Metamorph software (Molecular Devices Japan, Tokyo, Japan). FRET was performed in RoIs from 10 different cells. Three sets of images were acquired from each cell as follows: 1) Donor excitation-Donor emission, 2) Acceptor excitation-Acceptor emission, and 3) Donor excitation-Acceptor emission.

### Covalent cross-linking of TSHR and Western blot analysis

TSHR-expressing cells were cultured in 10 mM carbonate buffer containing 0.15 M NaCl with or without 1 mU/ml TSH for 1 h. Dimethyl suberimidate (DMS) (Pierce, Rockford, IL) was added to the medium at a final concentration of 2 mg/ml, followed by further incubation for 30 min. After addition of Laemmli buffer, samples were electrophoresed on 12.5% SDS-polyacrylamide gels, transferred to nitrocellulose membrane, and stained with TSHR polyclonal antibody (14450-1-AP; ProteinTech Group, Inc., Chicago, IL) at a dilution of 1∶500.

### Assay for thyroid hormones, ^125^I-TSH binding activity and TSH binding activity

Serum-free T3 and free T4 levels were assayed using the ECLusis system (Roche Diagnostic Co., Tokyo, Japan). Mouse TSH was assayed using the Rat TSH ELISA kit (AKRTS-010; Shibayagi, Gunma, Japan). ^125^I uptake by thyroid glands was measured by administering 10^4^ Bq of ^125^I-Na (GE Healthcare, Tokyo, Japan) into the peritoneal space. After 24 h, mice were anesthetized with pentobarbital, the thyro-tracheal unit was resected, and radioactivity was measured with a gamma counter (Autowell Gamma System, ARC-380; Aloka, Tokyo, Japan). ^125^I-TSH binding activities in the cells were determined using the methods of Mizutori et al. [9] with ^125^I-bovine TSH (Cosmic Co., Tokyo, Japan) and bovine TSH (Sigma-Aldrich, Inc., St. Louis, MO).

### Statistical analysis

Statistical analysis was carried out by one-way ANOVA and Student's t-test.

## Results

### Function of and morphological changes in thyroid glands from Tshr^hyt/wild^ mice

We previously reported that rectal temperature is a sensitive marker of thyroid status in mice [8]. We noticed that rectal temperature of Tshr^hyt/wild^ mice at room temperature (36.9±0.20°C) was significantly lower than that of wild-type mice (38.1±0.19°C) (p<0.001). When Tshr^hyt/wild^ mice were exposed to cold (4°C), rectal temperature rapidly dropped to 30.7±0.77°C at 120 min, whereas that of wild-type mice was 37.6±0.71°C (p<0.001) (Table 1), suggesting that Tshr^hyt/wild^ mice are in an overt hypothyroid state.

**Table 1:** Thyroid status in C.RF-Tshr^hyt/wild^ mice.

Mouse	Rectal temp (room, °C)	Rectal temp (cold, °C)	Lobe diameter (mm)	fT3 (pg/ml)	fT4 (ng/dl)	TSH (ng/ml)	^125^I-uptake (10^6^ cpm)	Anti-Tg (IU/ml)
W1	38.5	38.4	3.5	1.96	2.11	2.1	3.67	<0.3
W2	37.8	36.5	3.3	2.39	1.76	2.7	3.80	<0.3
W3	38.7	37.3	3.5	2.24	1.62	2.5	3.74	<0.3
W4	38.2	37.3	3.6	2.18	1.92	2.1	4.00	<0.3
W5	38.0	37.2	3.75	2.42	1.91	3.0	3.86	<0.3
W6	38.4	38.1	3.53	2.41	2.12	2.4	3.72	<0.3
W7	38.4	38.4	3.75	2.08	1.73	2.1	3.83	<0.3
mean	38.1±0.19	37.6±0.71	3.65±0.07	2.24±0.07	1.88±0.07	2.4±0.12	3.80±0.4	
Ht 1	36.5	25.9	2.5	2.00	0.92	18	1.90	<0.3
Ht 2	37.2	30.0	2.9	1.90	1.20	21.1	2.21	<0.3
Ht 3	35.8	32.9	3.0	1.92	1.12	15.5	2.34	<0.3
Ht 4	37.2	32.6	2.8	1.97	1.58	10	3.13	<0.3
Ht 5	37.3	32.6	2.6	1.75	1.17	19.2	3.04	<0.3
Ht 6	37.5	30.4	2.4	1.93	1.43	7.2	2.95	<0.3
mean	36.9±0.20**	30.7±0.77**	2.57±0.08**	1.91±0.04*	1.24±0.09**	13.8±1.3**	2.60±0.2**	

* : p<0.05,

** : p<0.001.

**Figure 1:**
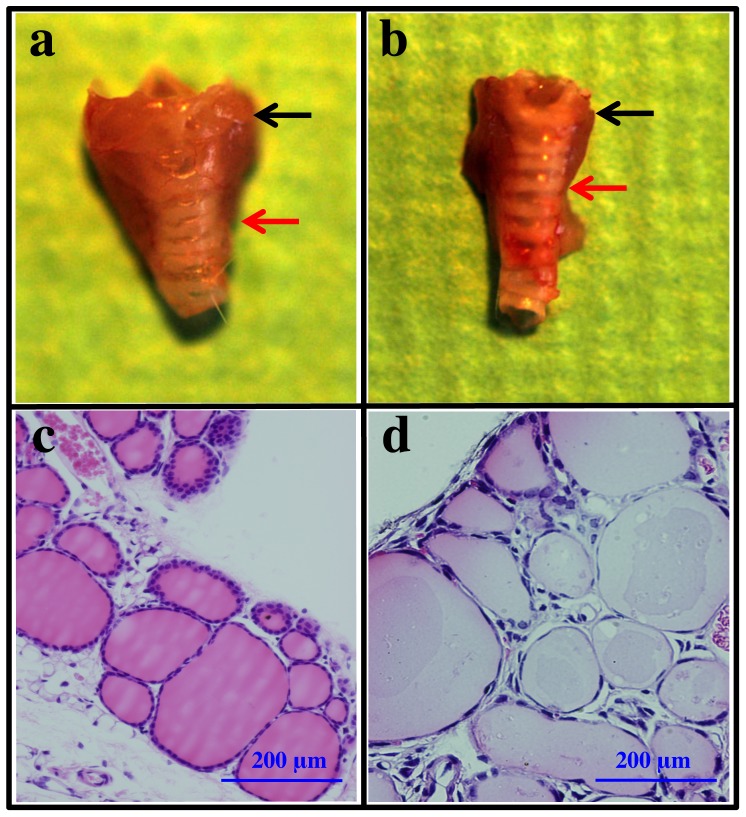
Thyroid glands from C.RF-Tshr^wild/wild^ and Tshr^wild/hyt^ mice. Macroscopic views of thyroid glands from 12-week-old C.RF-Tshr^wild/wild^ (a) and Tshr^wild/hyt^ (b) mice observed under a stereomicroscope (Olympus SZX7). Black arrows indicate the upper poles, and red arrows indicate the lower poles of the left lobe of the thyroid gland. Histology on high magnification in C.RF-Tshr^wild/wild^ (d) and Tshr^wild/hyt^ (e) mice. Tissues were fixed with 4% formaldehyde and embedded in paraffin. Sections (5 µm) were stained with hematoxylin-eosin. Bars indicate 200 µm.

Thyroid glands in Tshr^hyt/wild^ mice were smaller than those in wild-type mice (Fig. 1a, b). When the mean maximum diameter of the left and right lobes was measured, the value in Tshr^hyt/wild^ mice (2.57±0.08 mm, n = 6) was significantly smaller than that in wild-type mice (3.65±0.07 mm, n = 7) (Table 1). Free T4 and free T3 levels in Tshr^hyt/wild^ mice were significantly lower than those in wild-type mice, and serum TSH levels in the former were higher than those in the latter. ^125^I uptake activity of Tshr^hyt/wild^ mice was significantly lower than that in wild-type mice. Anti-thyroglobulin autoantibodies were not detected in either Tshr^hyt/wild^ mice or wild-type mice (Table 1).

Microscopically, thyroid glands in Tshr^hyt/wild^ mice consisted of larger follicles with thin thyroid epithelial cells (Fig. 1c, d). Collins and Capen reported that thyroid follicular cells became more columnar and follicular lumens became smaller after TSH treatment [10]. These results, as well as thyroid functions, suggest that despite the high TSH concentrations in sera, TSH signals in thyroid glands of the Tshr^hyt/wild^ mice are impaired, which may lead to overt hypothyroidism.

### Receptor function of P556L-TSHR (TSHR(M))

**Figure 2:**
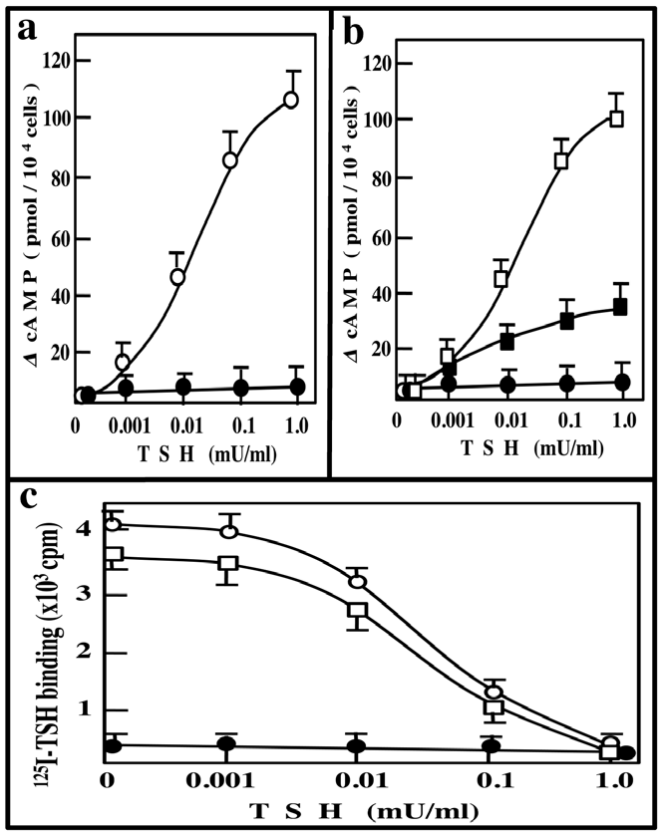
cAMP response to TSH and ^125^I-binding activities of HEK 293 cells expressing wild-type TSHR and/or mutated TSHR. (a) Cyclic AMP levels in HEK 293 cells expressing TSHR(W) or TSHR(M) following TSH stimulation. TSH was added to culture medium of HEK 293 cells transfected with 0.5 µg of pcDNA3-TSHR(W) (○-○) or the same amount of pcDNA-TSHR(M) (•-•). After 30 min, cellular cAMP levels were assayed. Data are means ± S.E. of triplicate assays. (b) Cyclic AMP levels in HEK 293 cells expressing TSHR(W) and TSHR(M) following TSH stimulation. TSH was added to culture medium of HEK 293 cells transfected with 0.5 µg of pcDNA3-TSHR(W)+the same amount of pcDNA (□-□), 0.5 µg of pcDNA3-TSHR(W)+the same amount of pcDNA-TSHR(M) (▪-▪) or 0.5 µg of pcDNA-TSHR(M)+the same amount of pcDNA (•-•). After 30 min, cellular cAMP levels were assayed. Data are means ± S.E. of triplicate assays. (c) ^125^I-binding activities of HEK 293 cells expressing wild-type TSHR and/or mutated TSHR. HEK 293 cells were transfected with 0.5 µg of pcDNA3-TSHR(W)+same amount of pcDNA (○-○), 0.5 µg of pcDNA3-TSHR(W)+the same amount of pcDNA-TSHR(M) (□-□) or 0.5 µg of pcDNA-TSHR(M)+the same amount of pcDNA (•-•). After 72 h of culture, TSH binding activities were assayed using ^125^I-bovine TSH. Data are means ± S.E. of triplicate assays.

In order to clarify the mechanisms of hypothyroidism in Tshr^hyt/wild^ mice, we studied the effects of TSHR(M) on the function(s) of wild-type TSHR (TSHR(W)). TSH dose-dependently increased cAMP production in HEK 293 cells expressing TSHR(W), but had no effect in HEK 293 cells expressing TSHR(M) (Fig. 2a), as reported previously by Gu et al. [4]. When equal amounts of pcDNA-TSHR(W) (0.5 µg) and pcDNA-TSHR(M) (0.5 µg) were transfected into HEK 293 cells, cAMP production in response to TSH decreased to about 20% of that in HEK 293 cells transfected with TSHR(W) (0.5 µg) and pcDNA (0.5 µg) (Fig. 2b). Figure 2c shows the ^125^I-TSH binding activities of these cells. We observed high-affinity TSH binding activity in HEK 293 cells expressing TSHR(W) (Ka = 35 µU/ml), and no binding was observed in HEK 293 cells expressing TSHR(M). Maximal TSH binding and affinity for TSH in HEK 293 cells co-expressing TSHR(W) and TSHR(M) (Ka = 42 µU/ml) were nearly the same as in those expressing TSHR(W) (Fig. 2c). These results suggest that TSHR(M) has a dominant negative effect on TSHR(W) functions.

### Effects of TSHR(M) on monomer-polymer formation of TSHR

**Figure 3:**
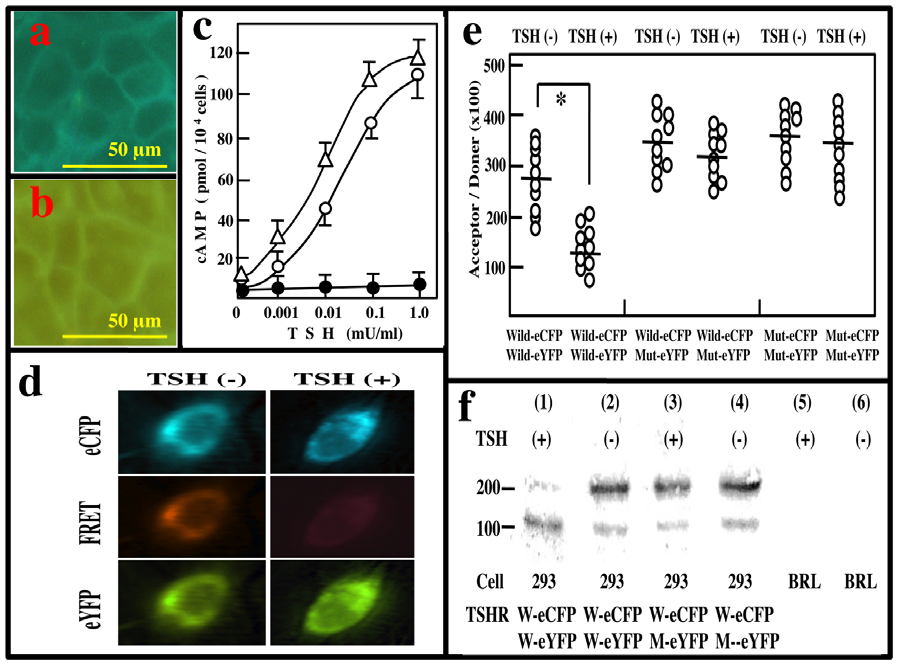
Interaction between TSHR(W) and TSHR(M). (a, b) Localization of TSHR(W)-eCFP and TSHR(M)-eYFP in HEK 293 cells. HEK293 cells were transfected with 0.5 µg of pcDNA3-TSHR(W)-eCFP (a) or 0.5 µg of pcDNA3-TSHR(M)-eYFP, and after 48 h, fluorescence were observed with an Olympus IX71 fluorescence microscope (×200). Bars indicate 50 µm. (c) cAMP responses of tagged (TSHR(W)-eCFP) and untagged TSHR (TSHR(W)). TSH was added to culture medium of HEK 293 cells transfected with 0.5 µg of pcDNA3-TSHR(W) (Δ-Δ) or the same amount of pcDNA-TSHR(W)-eCFP (○-○). 0.5 µg of pcDNA3 was also transfected into HEK293 cells (•-•). After 30 min, cellular cAMP levels were assayed. Data are means ± S.E. of triplicate assays. (d) FRET image of TSHR(W)-eCFP cells fused to TSHR(W)-eYFP. HEK 293 cells were transfected with 0.5 µg of pcDNA-TSHR(W) and the same amount of pcDNA-TSHR-eYFP, and were grown in medium for 72 h for live cell imaging. Left panels show fluorescence of donor, eCFP (upper panel), acceptor, eYFP (lower panel) and FRET image (middle panel) in the absence of TSH. Right panels show donor (upper panel), acceptor (lower panel) and FRET image (middle panel) in the presence of 1 mU/ml TSH. (e) Acceptor/Donor ratio in FRET assay from HEK 293 cells expressing TSHR(W or M)-eCFP and TSHR(W or M)-eYFP in the presence or absence of TSH. Acceptor/donor ratio was measured by Metamorph software in RoIs from 10 different cells expressing TSHR(W)-eCFP and TSHR(W)-eYFP, TSHR(W)-eCFP and TSHR(M)-eYFP, and TSHR(M)-eCFP and TSHR(M)-eYFP in the presence or absence of 1 mU/ml TSH. Bars indicate means ± S.E. *: p<0.01. (f) Cross-linking between TSHR(W) and TSHR(M) in the presence or absence of TSH. HEK 293 cells expressing TSHR(W)-eCFP and TSHR(W)-eYFP, and TSHR(W)-eCFP and TSHR(M)-eYFP were cultured in the presence or absence of TSH for 24 h, and dimethyl suberimidate was added to the medium at a final concentration of 2 mg/ml, followed by further incubation for 30 min. Proteins were electrophoresed, transferred onto nitrocellulose filter, and TSHR was detected with anti-TSHR antibody. BRL: BRL-3A, rat liver cells.

In order to study the effects of P556L mutation in the fourth transmembrane domain of TSHR on monomer-polymer formation, we prepared plasmids that express TSHR(W) or TSHR(M) tagged with eCFP or eYFP at the C-terminals (pcDNA-TSHR(W)-eCFP, pcDNA-TSHR(W)-eYFP, pcDNA-TSHR(M)-eCFP, and pcDNA-TSHR(M)-eYFP). When pcDNA-TSHR(W)-eCFP or pcDNA-TSHR(M)-eYFP was transfected into HEK 293 cells, both eCFP and eYFP signals were observed in transmembrane areas (Fig. 3a,b), as reported previously [3]. TSHR(W)-eCFP elicited cAMP response, as compared with the untagged receptor (Fig. 3c). Basal activities of HEK 293-TSHR(W) and HEK 293-TSHR(W)-eCFP were 9.3±1.5 and 8.2±1.1 pmols cAMP/10^4^ cells, respectively, and maximal response of the former was 115±9.1 and the latter was 105±11 pmols cAMP/10^4^ cells. Half maximal effective concentrations (EC50) of the tagged and the untagged receptors were 18 and 35 µU/ml, respectively. These results indicate that the tagged receptor is functional.

When pcDNA-TSHR(W)-eCFP (0.5 µg) and pcDNA-TSHR(W)-eYFP (0.5 µg) were co-transfected into HEK 293 cells, we observed fluorescence resonance energy transfer (FRET) from eCFP to eYFP in the absence of TSH, but not in the presence of TSH (Fig. 3d). Mean acceptor/donor ratio in ten HEK 293 cells expressing TSHR(W)-eCFP and TSHR(W)-eYFP in the absence of TSH was significantly (p<0.001) larger than that in the presence of TSH (Fig. 3d). However, we obtained high FRET in HEK 293 cells expressing TSHR(W)-eCFP and TSHR(M)-eYFP, and TSHR(M)-eCFP and TSHR(M)-eYFP, regardless of whether TSH was present (Fig. 3e). These results suggest that TSHR(M) loses the ability to dissociate into monomers in the presence of TSH.

The protein-protein interactions of TSHR were also observed in a cross-linking experiment. HEK 293 cells expressing TSHR(W)-eCFP and TSHR(W)-eYFP were cultured with or without TSH, followed by cross-linking with dimethyl suberimidate (DMS). Anti-TSHR antibody recognized two bands at 250 and 120 kDa. In the presence of TSH, the 125-kDa band was dominant, but in the absence of TSH, the 250-kDa band was stained more strongly than the 120-kDa band. When HEK 293 cells expressing TSHR(W)-eCFP and TSHR(M)-eYFP were cultured and cross-linking agent was added to the medium, the 250-kDa band was dominant, irrespective of the presence or absence of TSH (Fig. 3f).

## Discussion

The hyt/hyt recessive trait mouse, which provides a reproducible model for studying inherited hypothyroidism, was initially described by Beamer et al. [11]. Hyt/hyt homozygous animals can easily be differentiated by their obviously smaller size, and few differences in size and in serum thyroid hormone levels are observed between the hyt/wild and the wild/wild mice. Thus, many studies, even after identification of a point mutation in the TSHR gene [3], have used hyt/hyt mice as hypothyroid animals, and hyt/wild and the wild/wild groups as euthyroid mice [12], [13].

In the present study, we determined the genotypes of Tshr^hyt/hyt^, Tshr^hyt/wild^ and Tshr^wild/wild^ mice by direct sequencing of the gene, and found that serum-free T4 levels in Tshr^hyt/wild^ mice are significantly lower than in Tshr^wild/wild^ mice. In addition, rectal temperature, size of the thyroid gland, and ^121^I uptake activity of Tshr^hyt/wild^ mice indicated overt hypothyroidism.

However, these results are inconsistent with the report of TSHR KO mice by Marians et al. [2]. TSHR^KO/KO^ mice show severe hypothyroidism with no detectable thyroid hormone, and die within 1 week. No significant differences in thyroid hormone and TSH, or in growth and development, are observed in TSHR^KO/wild^ mice and TSHR^wild/wild^ mice. As P556L TSHR has been reported to properly integrate into the plasma membrane [4], we hypothesized that this mutant receptor has a dominant negative effect on the wild-type receptor. Indeed, a co-expression study of mutant and wild-type receptors in 293 cells demonstrated that mutant receptors impaired TSH-induced cAMP production by wild-type receptors.

Using TSHR differentially tagged with RFP and YFP, Latif et al. previously demonstrated by FRET that TSH-induced activation of the TSHR would result in disruption of the dimer [14]. However, Urizar et al. were unable to confirm these results in a bioluminescence resonance energy transfer (BRET) experiment [15]. We obtained FRET from TSHR(W)-eCFP to TSHR(W)-eYFP only in the absence of TSH, and from TSHR(W)-eCFP to TSHR(M)-eYFP in the presence of TSH. Therefore, our results support the hypothesis of Latif et al. and suggest that the hyt mutation, the P556L mutation in the fourth transmembrane domain, interferes with TSH-induced dissociation of dimer to monomer. As a consequence thereof, our results support the hypothesis proposed by Latif et al. [14] that only monomers should contribute to G protein activation and the release of second messengers.

Recently, Latif et al. also reported that a tyrosine residue, Y116, in the extracellular domain of TSHR plays an important role in stabilizing multimer formation of the receptor [16], but the present data suggest that the fourth transmembrane domain also confers monomer-multimer dissociation of TSHR.

In addition to entrapment of wild-type TSHR in endoplasmic reticulum by the mutant receptor, which induces TSH resistance in humans [17], this is the first report linking dominant negative mutations of a G protein-coupled receptor integrating in plasma membrane to an abnormal endocrine phenotype in mammals, and provides an explanation for hypothyroidism in C.RF-Tshr^hyt/wild^ mice.
